# An Alkaline-Acid Glycerol Electrochemical Reformer for Simultaneous Production of Hydrogen and Electricity

**DOI:** 10.3390/nano12081315

**Published:** 2022-04-12

**Authors:** Fernando M. L. Amorim, Rudy Crisafulli, José J. Linares

**Affiliations:** 1 Institute of Chemistry, Federal University of Goiás, Campus Samambaia, Avenida Esperança s/n, Goiania 74690-900, Brazil; fernando.migueldelino@gmail.com; 2 Institute of Chemistry, University of Brasilia, Campus Universitário Darcy Ribeiro, Brasilia 70910-900, Brazil; rudycrisafulli@gmail.com

**Keywords:** glycerol, hydrogen, electrolysis, alkaline-acid, energy, spontaneity

## Abstract

This study shows the results, for the first time, of an glycerol alkaline-acid electrolyzer. Such a configuration allows spontaneous operation, producing energy and hydrogen simultaneously as a result of the utilization of the neutralization and fuel chemical energy. The electroreformer—built with a 20 wt% Pd/C anode and cathode, and a Na^+^-pretreated Nafion^®^ 117—can simultaneously produce hydrogen and electricity in the low current density region, whereas it operates in electrolysis mode at high current densities. In the spontaneous region, the maximum power densities range from 1.23 mW cm^−2^ at 30 °C to 11.9 mW cm^−2^ at 90 °C, with a concomitant H_2_ flux ranging from 0.0545 STP m^−3^ m^−2^ h^−1^ at 30 °C to 0.201 STP m^−3^ m^−2^ h^−1^ at 90 °C, due to the beneficial effect of the temperature on the performance. Furthermore, over a chronoamperometric test, the electroreformer shows a stable performance over 12 h. As a challenge, proton crossover from the cathode to the anode through the cation exchange Nafion^®^ partially reduces the pH gradient, responsible for the extra electromotive force, thus requiring a less permeable membrane.

## 1. Introduction

On the ever-closer energy scenario dominated by renewable energies, energy vectors are expected to play a key role in order to match offer and demand [1]. In this sense, hydrogen has emerged as an attractive candidate due its high mass energy density, environmentally friendly character (as H_2_O is the combustion product), and its generation from several sources—such as biogas, natural gas, and water electrolysis, among others [2,3]. Hydrogen is not freely available in nature, being produced by chemical processes. According to the International Energy Agency, in 2020, H_2_ production was 90 Mt, mostly from fossil fuels—such as naphtha reforming (21%), steam natural gas reforming (59%), and coal gasification (19%)—as the most notable sources. Such a scenario mandates changes in the search for a more sustainable economy. Eco-friendly routes for clean hydrogen production involve the so-called ‘blue hydrogen’ (produced from natural gas with carbon capture, 0.7% of the overall production) and ‘green hydrogen’, mainly from water electrolysis (0.03%), with zero dependence on fossil fuels [4].

Water electrolysis, as the cleanest method to produce hydrogen, presents some advantages, such as the possibility of using renewable energies for powering the electrolyzer and the high purity of the hydrogen produced compared to fossil fuel-based processes, where purification stages are required [5,6,7]. As a counterpoint, hydrogen electrolysis is expensive. According to the IEA, hydrogen from natural gas costs approximately 0.5–1.7 USD/kg^–1^, whereas green hydrogen is around 3–8 USD/kg^–1^. The electricity requirements for water electrolysis notably impact the cost of production of H_2_ (up to 75%). In this way, the energy demand for this system is one of the crucial goals in making electrolysis competitive.

The main reason for the high electricity requirement of water electrolysis is the high electromotive force (emf), whose theoretical value is −1.23 V. In practical conditions, required voltages in the range of 1.6–2.0 V are necessary, resulting in an overall energy demand of 50 kWh kg^–1^ of H_2_ [8]. One of the alternatives to overcome this is the substitution of the water oxidation half-reaction (standard redox potential 1.23 V vs. normal hydrogen energy, NHE) by organic molecules whose standard redox potentials are significantly lower (in the range of 0–0.1 V vs. NHE for most short-chain alcohols). Through this approach, the energy demands can be, on average, halved [2,9,10].

In parallel, another approach to reducing the electricity demand is the utilization of an alkaline-acid electrochemical cell. This approach has been already explored for direct ethanol fuel cells [11,12] and direct glycerol fuel cells [13]. It has also been successfully applied to water electrolysis (amphoteric water electrolysis) [14,15,16,17,18], a Zn–H_2_ fuel cell [19], and for urea electrolysis coupled with H_2_ production [20,21]. Furthermore, some studies, based on the principle of an alkaline-acid configuration, developed an acid–base electrochemical flow battery [22,23,24]. The common principle of these configurations is the operation under different pHs at the anode and cathode. Two advantages arise from this arrangement: (1) in general, oxidation reactions performing better under alkaline medium, whereas reduction reactions are more active in an acidic medium, e.g., the hydrogen evolution reaction; (2) the neutralization electromotive force that provides additional energy for the electrolyzer.

For the glycerol alkaline-acid electroreformer, the corresponding electrochemical reactions are collected in Equations (1) and (2), whose theoretical voltage are also included [13,25]:

Alkaline anode: C_3_H_8_O_3_ + 20 OH^−^ → 3 CO_3_^2−^ + 14 H_2_O + 14 e^−^
E^0^ = −0.82 V vs. NHE (1)

Acidic cathode: 2 H^+^ + 2 e^−^ → H_2_
E^0^ = 0.00 V vs. NHE (2)

Overall: C_3_H_8_O_3_ + 6 OH^−^ + 14 H (3)

The half-cell potentials depict a cell potential difference of 0.82 V, indicating that the system could even be spontaneous. This may be possible thanks to the pH gradient between the anode and the cathode (≈14), which allows an extra emf associated to the ΔpH of 0.828 V [14,25].

In this way, we propose the development of a glycerol electrochemical reformer based on operation under alkaline conditions at the anode and acidic at the cathode. With this, it will be possible to take the advantage of the favorable pH gradient between the anode and the cathode to gain emf (0.828 V), allowing for the spontaneous operation of a range of current densities whilst simultaneously producing hydrogen. The electroreformer is based on the use of glycerol, a green alcohol obtained as by-product from biodiesel synthesis, whose valorization can turn the biodiesel economically more attractive. A Pd/C electrocatalyst, prepared by a sodium formate chemical reduction, was used as anode and cathode catalyst. Palladium nanoparticles supported on carbon black are known to be very active for glycerol electro-oxidation reaction (GEOR) [10,26,27], as well as for hydrogen evolution reaction (HER) in acidic medium [28]. Initial preliminary CV tests were carried in a three-electrode glass cell, showing the potential of this approach for the spontaneous operation. The real application was displayed in a single-cell alkaline-acidic glycerol electroreformer based on a Nafion^®^ 117 membrane pre-treated with NaOH. Different temperatures were applied to evaluate the influence of this parameter, along with preliminary stability tests, also assessing the initial and final pH of the anolyte and catholyte to assess the possible crossover of ionic species.

## 2. Materials and Methods

### 2.1. Chemicals

Sodium tetrachloropalladate (Na_2_PdCl_4_, 98 wt% purity) and the Nafion^®^ 117 cationic exchange membrane were purchased from Sigma-Aldrich (Jurubatuba, Brazil). Sodium hydroxide (97 wt% purity, P.A.-ACS), sulfuric acid (98 wt% P.A.), formic acid (85 wt% purity, P.A.-ACS), glycerol (99.5 wt% purity, P.A.-ACS), and 2-propanol (99.5 wt% purity, P.A.-ACS) were acquired from Dinâmica (São Paulo, Brazil). For titration, a solution 1 mol L^−1^ NaOH (Sigma-Aldrich, Titripur^®^) and 1 mol L^−1^ HCl (Sigma-Aldrich, Titripur^®^) were used for acid and alkali quantification. Vulcan XC-72R was purchased from Cabot Corporation (Boston, Massachusetts, USA). Nafion^®^ (5 wt% in a mixture of aliphatic alcohols) was acquired from IonPower (New Castle, DE, USA).

### 2.2. Preparation of the Pd/C Catalyst

The Pd/C catalyst was prepared by the sodium formate reduction method. In a typical procedure for preparing 100 mg of 20 wt% Pd/C, 100 mL of a 0.01 mol L^–1^ sodium formate solution was prepared. The pH of the solution was increased to 13 by the addition of some droplets of a 4 mol L^–1^ NaOH solution. Next, 80 mg of carbon was added and sonicated for 30 min with the aid of an ultrasonic tip (Sonics Vibra-Cell VC 505, Biovera, Rio de Janeiro, Brazil). Afterwards, the reaction mixture was poured into a jacketed reactor and heated to 80 °C in a thermostatic bath. In parallel, 55.3 mg of Na_2_PdCl_4_ was dissolved in 8 mL of water in an ultrasonic bath. The Na_2_PdCl_4_ solution was divided into three portions of the same volume and added dropwise with the aid of a burette. After completing the addition of the Pd solution, the temperature was maintained at 80 °C for 1 h and the system maintained under stirring for 12 h. The Pd/C electrocatalyst was then filtered, washed thoroughly with boiling water, and left to dry at 70 °C for 12 h. The obtained material was then ground to obtain a fine powder and stored under inert atmosphere for future use.

### 2.3. Physico-Chemical Characterization

To verify the final metal loading, EDX analysis was carried out using a JEOL JSM 66,100 scanning microscope with 30-kV acceleration voltage. The XRD analysis was carried out on a D/MAX-B Geiger-flex diffractometer (Rigaku, Tokyo, Japan) using the Cu-K_α1_ (0.154 nm), between 2θ angles of 20° and 90°, at 0.5° min^–1^ and steps of 0.05°. The average crystallite size was estimated by Scherrer’s equation [29]. TEM images were obtained from a JEOL 2100 microscope at 200 kV at the LabMic (Laboratório Multiusuário de Microscopia de Alta Resolução, Univ. Federal de Goiás, Goiânia, Goiás, Brazil). From the images, the average particle size (D) was estimated from Equation (4) [30], where n_i_ is the number of particles whose size is D_i_:

(4) $$D=\frac{\sum_{i}n_{i}D_{i}}{\sum_{i}n_{i}}$$

### 2.4. Electrochemical Measurement in the Three-Electrode Glass Cell

The initial electrochemical characterization of the prepared Pd/C was carried out by cyclic voltammetry (CV) in supporting electrolytes: 1 mol L^–1^ NaOH for GEOR and 0.5 mol L^–1^ H_2_SO_4_ for the HER. Next, the GEOR measurements were carried out by preparing a solution of 1 mol L^–1^ glycerol in 1 mol L^–1^ NaOH. In the case of the HER, the same H_2_SO_4_ solution was used. The working electrode was prepared by dispersing 4 mg of Pd/C in 1 mL of 2-propanol and 10 µL of Nafion^®^ emulsion in an ultrasonic bath for 30 min. With the aid of an automatic pipette, 10 µL was deposited onto a circular reticulated vitreous carbon support (diameter = 5 mm) inserted in a Teflon rod. The counter-electrode was a platinized platinum mesh, whereas the reference electrode for alkaline experiments was a Hg/HgO electrode and Ag/AgCl for acidic medium. A μ-Autolab (model Type III) potentiostat/galvanostat was coupled to a personal computer. General-Purpose Electrochemical System (GPES) software was used for the electrochemical measurements. Alkaline blank CVs were performed between –0.926 and 0.474 V vs. Hg/HgO at a scan rate of 0.05 V s^–1^. The same window was used for GEOR with a reduced scan rate of 0.005 V s^–1^. Acid blank CVs were recorded between –0.15 and 1 V vs. Ag/AgCl, whereas for the HER, the voltage was cycled between 0 and −0.4 V vs. Ag/AgCl. Curves were repeated until a stable voltammogram shape was obtained. For comparison purposes, oxygen evolution reaction (OER) was also evaluated with potential limits between −0.8 and 0.9 V vs. Hg/HgO.

Electrochemical reforming tests were carried out in a 4-cm^2^ single-cell electrochemical reformer. Briefly, the system is composed of two graphite monopolar plates with parallel channels of 1 mm thickness and depth, and 2 cm length with 10 channels. Stainless-steel end plates were used as current collectors and mechanical support for closing and tightening the single cell. In order to control the temperature, a thermocouple was inserted within the graphite plate of the anode, connected to a temperature controller (Novus N1020, Canoas, Brazil), whose controlling action was executed onto heating rods inserted within the stainless-steel end plates. Two peristaltic pumps (EX-P4203, Exatta, Palhoça, Brazil) were used to supply the fuel (1 mol L^–1^ of glycerol in 4 mol L^–1^ NaOH solution, established in a previous study [31]) and catholyte (1 mol L^–1^ H_2_SO_4_ solution) solutions at 1 mL min^−1^. More details can be found elsewhere [32]. For the preparation of the electrodes, a slurry containing the required mass of catalyst (2 mg cm^–2^ of Pd in the anode and 0.5 mg cm^–2^ of Pd in the cathode), Nafion^®^ emulsion at 10 wt% normalized with respect to the amount of carbon (IonPower, USA), and a mixture of 2-propanol and water as the solvent were used. The slurry was paint-brushed onto a carbon cloth diffusion layer (Zoltek, Bridgeton, MO, USA). As membrane electrolyte, a Nafion^®^ 117 membrane was used, previously soaked in 1 mol L^−1^ NaOH at 80 °C for 1 h. Next, the membrane was thoroughly washed with water to exchange the H^+^ with Na^+^ ions [33]. The polarization curves were recorded with a AUTOLAB PGSTAT 302 N potentiostat/galvanostat (Metrohm Autolab BV, Utrecht, The Netherlands) in galvanodynamic mode from zero current up to that corresponding to a voltage corresponding to an electrochemical window of 1.4 V, at a scan rate of 0.1 mA s^–1^. Hydrogen was collected with an inverted burette system over the whole polarization curves. Equation (4) was used to estimate the Faradaic efficiency by comparison of the measured H_2_ (V_H2,exp_, normalized to STP conditions, 10^5^ Pa and 273 K) and the theoretical one, estimated by the denominator of Equation (5). The parameter I(t) is the recorded current, F is the Faraday constant (96,485 C mol^−1^), t is the time, and 22,711 is the volume in mL of 1 mol of H_2_ in STP conditions.

(5) $$\mathrm{Efficiency}\left(\%\right)=\frac{{100V}_{H2,\exp}\left(\mathrm{mL}\right)}{\frac{\int_{0}^{t}I\left(t\right)\mathrm{dt}\;\left(C\right)}{2F\left({C\;\mathrm{mol}}^{-1}\right)}22,711\left({\mathrm{mL}\;H}_{2}{\mathrm{mol}}^{-1}{\;H}_{2}\right)}$$

Finally, galvanostatic experiments were carried out to verify the stability of the electrolyzer at the current density where the system presented the maximum power density in the spontaneous mode. Furthermore, the initial and final OH^−^ and H^+^ concentrations were assessed by titration with the standard solution of HCl and NaOH. The volumes of both anolyte and catholyte were 50 mL.

## 3. Results and Discussion

Table 1 collects the results from EDX regarding the Pd loading in the prepared catalyst. As can be seen, the experimental value was very close to the nominal, corroborating the successful deposition of Pd nanoparticles (EDX spectrum in the Supplementary Materials, Figure S1). The results corresponding to the XRD patterns and TEM images are briefly presented and briefly discussed in Figures S2 and S3, with the typical patterns of the Pd fcc crystalline structure (Figure S2), as well as the presence of nanosized Pd particles distributed in a relatively homogeneous manner on the carbon support (Figure S3). Table 1 summarizes the main information extracted from the physico-chemical characterization.

**Figure 1:**
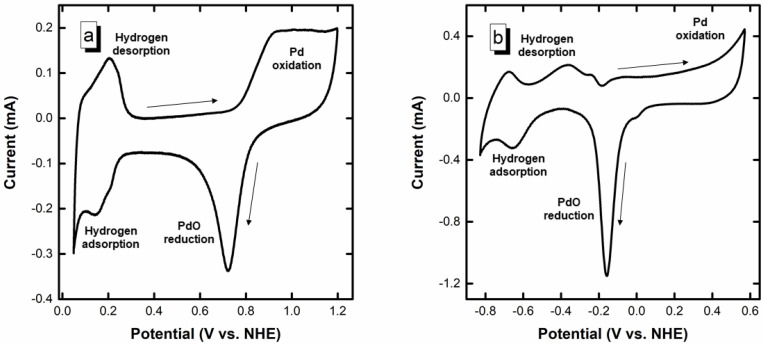
(**a**) Blank CV in 0.5 mol L^−1^ H_2_SO_4_, and (**b**) Blank CV in 1 mol L^−1^ NaOH. Arrows indicate the direction of the potential.

Figure 1 shows the blank voltammograms of the Pd/C electrocatalyst in 0.5 mol L^–1^ H_2_SO_4_ and 1 mol L^–1^ NaOH. The different regions of the voltammograms are also identified in Figure 1 according to Grdeń et al. [34]. The voltammogram in the acidic electrolyte (Figure 1a) shows the typical regions associated with the hydrogen adsorption/desorption between 0.05 and 0.3 V vs. NHE, whereas the formation and reduction of Pd oxide/hydroxide species are observed in the high potential region (>0.8 V vs. NHE in the forward scan and down to 0.6 V vs. NHE in the reverse scan) [35,36]. In NaOH (Figure 1b), the behavior is more complex and indeed involves the hydrogen adsorption/desorption region at low potentials combined with the formation of a premonolayer of adsorbed hydroxides (Pd-OH_ads_, approximately > –0.4 V vs. NHE) onto the palladium surface [36,37,38,39,40], completed by the formation of PdO at higher potentials (approximately > –0.15 V vs. NHE) [41]. In the cathodic scan, the maximum intensity of the reduction of the PdO can be observed at approximately –0.15 V vs. NHE. An initial tiny shoulder can be observed at 0.05 V in the PdO reduction peak. According to Grdeń et al. [34] and Chierchie et al. [42], the position of the Pd reduction peak can shift according to the density of package of the PdO formed, displacing to lower reduction potential the thicker and denser the PdO layer. Thus, the initial shoulder might be ascribed to the reduction of some thin PdO layer. From this, the electrochemically active surface area (EASA) can be estimated, considering an associated charge to a monolayer of PdO of 0.424 mC cm^–2^ [43]. The obtained average EASA was 66.3 ± 2.8 m^2^ g^–1^ Pd; for such an EASA, the average particle size is 7.5 ± 0.3 nm, according to Equation (6), where d is the average particle size and 11.9 × 10^7^ is the mass density of Pd in g m^−3^. Such higher particle size value can be attributed to some isolation of Pd nanoparticles in small pores of the carbon support or to the presence of some oxides that isolate the catalyst particles [44].

(6) $$d\left(\mathrm{nm}\right)=\frac{6\times 10^{9}\left({\mathrm{nm}\;m}^{-1}\right)}{11.9\times 10^{7}{g\;m}^{-3}\mathrm{EASA}\left(m^{2}g^{-1}\right)}$$

**Figure 2:**
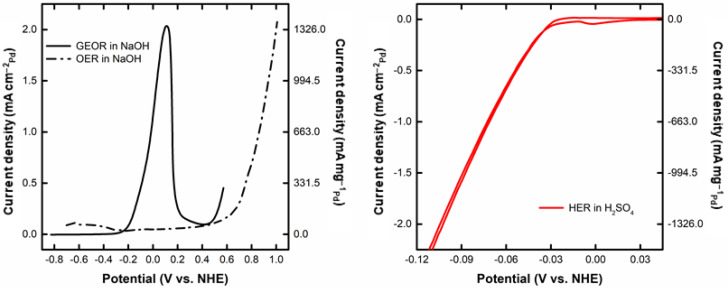
(**left**) CV for GEOR in 1 mol L^−1^ glycerol in 1 mol L^−1^ NaOH, OER in 1 mol L^−1^ NaOH, and (**right**) HER in 0.5 mol L^−1^ H_2_SO_4_ (measurement carried out in the three-electrode glass cell).

Figure 2 shows the corresponding voltammogram for GEOR and OER in NaOH (Figure 2 (left)), and for HER in H_2_SO_4_ (Figure 2(right)). Regarding the GEOR, the onset potential corresponds to a value of –0.24 V. In this region, the Pd surface is partially oxidated, providing the required oxygenated species for the GEOR [45,46,47]. The GEOR intensifies up to a maximum current of 2.03 mA cm^–2^ of Pd −1346 mA mg^–1^ of Pd, after which there is a drastic drop attributed to the massive coverage of the Pd surface by oxygenated species. At a potential above 0.4 V, there is a renewed increase in the current density, perhaps due to an activation of the PdO surface at high potentials. The activity of Pd at a potential of 1.299 V was recently observed (1.1 V vs. Ag/AgCl for ethylene oxidation to ethylene glycol [48]). In the reverse scan, a new peak appears at –0.05 V, corresponding to the reactivation of the Pd surface by the reduction of the PdO layer. The comparison with OER indicates the higher required potential for this process (>0.401 V vs. NHE [18]) compared to the GEOR (−0.82 V vs. NHE, Equation (1)). For instance, at a mass-normalized current density of 1 mA mg_Pd_^−1^, the potential for GEOR is −0.015 V vs. NHE, whereas for the OER the corresponding value is 0.82 V vs. NHE. Such differences are responsible for the reduced energy demand of the glycerol electrochemical reforming compared to water electrolysis [49], as already observed for ethanol electrolysis [50,51,52]. Figure 2 also displays the HER, showing a very low overpotential typical of this low-polarizable process [53]. This figure shows that it would be theoretically possible to operate this system spontaneously up to 0.64 mA cm^–2^ of Pd and 424.3 mA mg^–1^ of Pd (potential (E) of GEOR = potential (E) of HER = −0.063 V, at lower currents the emf (E_HER_−E_GEOR_) is positive, indicating spontaneity) thanks to the favorable pH gradient.

**Figure 3:**
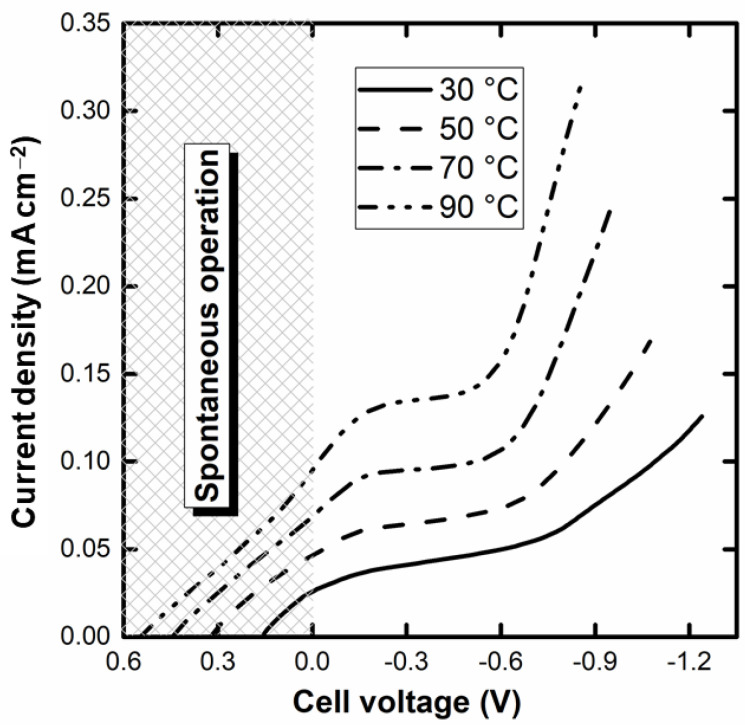
Current-voltage (E_HER_−E_GEOR_) curves of the glycerol alkaline-acid single-cell electrochemical reformer at different cell temperatures (voltage window of 1.4 V; anode: 4 mol L^−1^ KOH and 1 mol L^−1^ glycerol; cathode: 1 mol L^−1^ H_2_SO_4_; electrolyte: Na^+^-Nafion^®^ 117 membrane).

The polarization curves of the single-cell alkaline-acid glycerol electrochemical reformer are displayed in Figure 3. As can be seen, there is a voltage region in which it is possible to operate spontaneously from the extra emf coming from the pH gradient. This is a very remarkable feature and, to the authors’ knowledge, the first reported for alcohol electrochemical reforming. Up to 26, 49, 71, and 100 mA cm^–2^ at 30, 50, 70, and 90 °C, respectively, the electrolyzer operates with no energy requirement and, indeed, might be used as a fuel cell. The increase in the temperature enhances, as expected, the performance (and reduces the open circuit voltage) as a result of the activation of the GEOR and the HER, as well as the increase in the ionic conductivity of the membrane. The curves also show a horizontal asymptotic region for current at intermediate voltage, ascribed to the large coverage of the Pd surface by oxide species, after which the current again increases, giving the observations seen in Figure 2.

**Figure 4:**
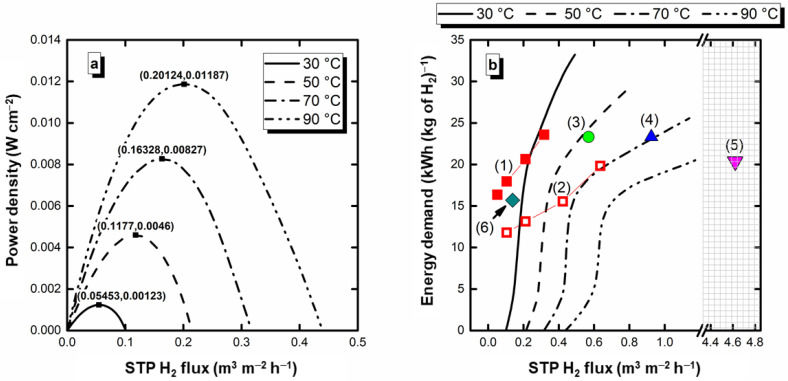
(**a**) Power vs. H_2_ flux of the glycerol alkaline-acid electrochemical reformer in the spontaneous region, and (**b**) Energy demand for the electrolysis mode region including comparison with other studies presented in the literature (see Table 2 for details about each entry).

Figure 4a shows the power density than can be drawn from the electroreformer vs. the H_2_ flux (estimated faradaic efficiencies above 99% from Equation (5)). As can be observed, in addition to the hydrogen production, energy from the existing pH gradient can be extracted from the electroreformer, with maximum H_2_ flux/power density pairs of (0.05453, 0.00123), (0.1177, 0.0046), (0.16328, 0.00827), and (0.20124, 0.01187) (STP m^3^ m^–2^ h^–1^, W cm^–2^) at 30, 50, 70, and 90 °C, respectively. These conditions may be depicted as optimal, as they combine hydrogen production with the maximum power density. Figure 4b shows the energy requirements in the electrolytic mode region, including the values found in other studies on Pd electrocatalysts [9,54,55,56,57]. As can be observed, compared to previous studies carried out by this research group with an alkaline electrochemical reformer, this alkaline-acid electroreformer results, in general, in a reduction in energy consumption, showing the advantage of adding the neutralization energy to the electroreformer (see Table 2 for details of the literature studies). Compared to the results of Prof. Vizza’s research group (entries 3 to 6 of Table 2), this system is more energy-consuming, indicating that there is still room for further improvements by, for instance, working on the electrocatalyst composition and architecture. Another barrier that requires improvement is the ionic conductivity. In this case, the Na^+^ cations are transported from the anode to the cathode [11,12,13], where their mobility is inferior to that of H^+^ within the Nafion^®^ membrane [58]. Thinner or alternative membranes could be considered to reinforce the advantages of the alkaline-acid electrolyzer, further boosting hydrogen and electricity production and the energy demand in the electrolytic region.

**Figure 5:**
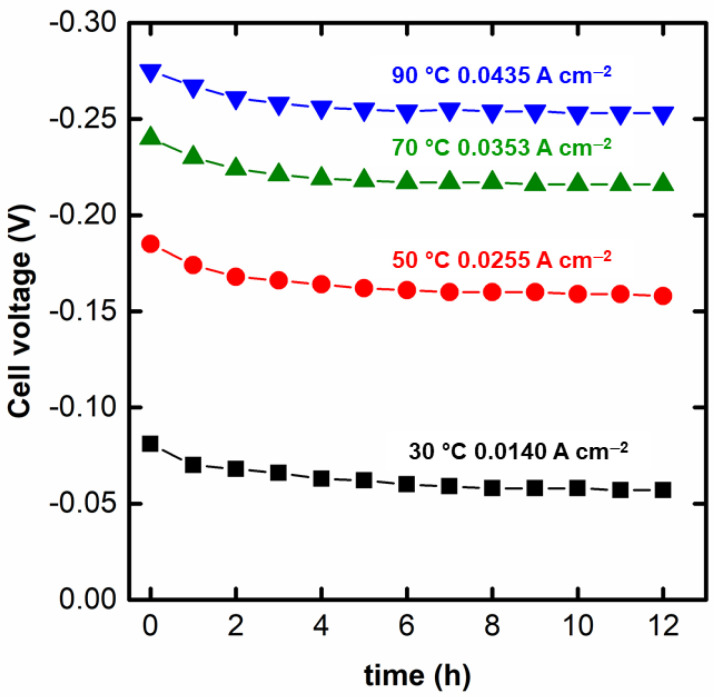
Preliminary stability test of the glycerol alkaline-acid electrochemical reformer for 12 h at the current density in which the maximum power density of the spontaneous region was achieved.

Finally, the stability of the system in the optimum condition for the simultaneous generation of hydrogen and electricity is displayed in Figure 5. The stability curves are presented for the current densities in which the maximum power density was drawn from the spontaneous region of the electrochemical reformer. As can be seen, for the four temperatures, the electroreformer presents a relatively stable performance with an initial decay attributed to the poisoning of the accumulation of adsorbates onto the Pd surface in the first 3 h [59]. After this, the Pd electrocatalyst seems to achieve a (pseudo)stationary state in which poisoning and recovery of the Pd surface seem to occur at similar rates. Further studies for longer times and more stressing conditions (dynamic load cycles) are recommended for a deeper study of this feature. Such conditions better match to the real demands of an electrolyzer [60]. In this sense, Table 3 collects the initial and final OH^−^ concentrations in the anolyte and H^+^ in the catholyte. It can be observed a decrease in both OH^-^ and H^+^ concentration due, primarily, to the consumption of these ions in the redox reactions (see Equations (1) and (2)), although non-electrochemical processes, such as proton crossover, could also contribute to the decrease in the concentration of H^+^ and OH^−^. An estimation of the final H^+^ concentration can be calculated from the application of the Faraday’s law (Equation (7)), considering the stoichiometry of Equation (2). The parameter $n_{H^{+}\mathrm{consumed}}$ represents the number of H^+^ consumed. In the case of the consumption of glycerol, the estimation of the OH^−^ consumption is not so straightforward given the complexity of the GEOR [10,54]. Several products with different OH^−^ consumption can be formed, which would turn necessary the complete product characterization, an issue that is currently under investigation. Table 4 presents a comparison of the theoretical final H^+^ concentration and the experimental one, along with the protons “consumed non-faradaically”.

(7) $$n_{H^{+}\mathrm{consumed}}=\frac{I\times t}{F}$$

As can be observed, the real final concentrations are lower than the theoretical ones predicted by the Faraday’s law. This behavior is explained by Wang et al. [16], Mundaray et al. [23], and Weng et al. [24] as a result of certain crossover of H^+^, leading to a loss of pH gradient and ionic species that participate in the redox process. In this manner, the efficiency of the system is somehow reduced. In fact, one of the challenges of the systems that utilize a pH gradient to generate a favorable emf is the development of materials with low protons and hydroxide anions permeability without excessively losing conductivity.

## 4. Conclusions

This work has demonstrated the possibility of simultaneously producing hydrogen and electricity from an alkaline-acid glycerol electroreformer (using a Na^+^–Nafion^®^ ion-exchange membrane and a Pd/C electrocatalyst). This very remarkable feature arises from the combined exploitation of the pH gradient—which allows an extra emf—and chemical (from the fuel) energy of the system. An increase in the temperature increases the maximum power density and the hydrogen rate, due to the improvement in the kinetics of the GEOR/alkaline and HER/acid, as well as the increase in the ionic conductivity of the electrolytic membrane. The system also presents a relatively stable performance for 12 h in a preliminary stability test at the current density in which the maximum power density is extracted from the cell, evidencing the feasibility of this novel approach. In contrast, attention should be given to the proton crossover through the membrane—the more intense, the higher the temperature—which presents a challenge for the development of membranes with low ionic permeability.

## Figures and Tables

**Table 1:** Main information extracted from the physico-chemical characterization.

Electrocatalyst	Experimental Pd Percentage from EDX	Average Crystallite Size by XRD (nm)	Average Particle Size by TEM (nm)
20% Pd/C	19.5 ± 0.8	5.0	5.5

**Table 2:** Information about the studies used for comparison purposes in Figure 4.

Reference Curves	Work	Anode	Fuel	Cathode	Catholyte	Membrane	Temperature
1	Costa Santos et al. [54]	2 mg cm^−2^ 20% Pd/C	1 mol L^−1^ glycerol and 4 mol L^−1^ KOH	1 mg cm^−2^ commercial 20% Pt/C	2 mol L^−1^ KOH	KOH-doped polybenzimidazole (PBI)	30 °C
2	Costa Santos et al. [54]	2 mg cm^−2^ 20% Pd/C	1 mol L^−1^ glycerol and 4 mol L^−1^ KOH	1 mg cm^−2^ commercial 20% Pt/C	2 mol L^−1^ KOH	KOH-doped polybenzimidazole (PBI)	90 °C
3	Bambagioni et al. [55]	Pd-(NiZn)/C on Ni foam anode (Pd loading 1 mg cm^−2^)	10 wt% glycerol in 2 mol L^−1^ KOH	Pt/C cathode on carbon paper (Pt loading 2 mg cm^−2^)	2 mol L^−1^ KOH	Tokuyama A006	40 °C
4	Bellini et al. [56]	Pd-(NiZn)/C on Ni foam anode (Pd loading 1 mg cm^−2^)	10 wt% glycerol in 2 mol L^−1^ KOH	Pt/C cathode on carbon paper (Pt loading 2 mg cm^−2^)	2 mol L^−1^ KOH	Tokuyama A201	Room temperature
5	Chen et al. [57]	Pd supported on titania nanotubes (Pd loading 1.7 mg cm^−2^)	2 mol L^−1^ glycerol and KOH	Pt/C on carbon cloth cathode (Pt loading 0.3 mg cm^−2^)	No liquid	Tokuyama A201	80 °C (the study also included 25 and 50 °C)
6	Bellini et al. [9]	Pd-CeO_2_/C (Pd loading of 1 mg cm^−2^) supported onto Ni foam	2 mol L^−1^ glycerol and KOH	Commercial 40% Pt/C (Pt loading 0.4 mg cm^−2^) on carbon cloth	Not specified	Tokuyama A201	60 °C

**Table 3:** Initial and final OH^−^ and H^+^ concentrations over the chronoamperometric.

Temperature (°C)	Current Density (A cm^−2^)	Anolyte		Catholyte	
		Initial OH^−^ Concentration (mol L^−1^)	Final OH^−^ Concentration (mol L^−1^)	Initial H^+^ Concentration (mol L^−1^)	Final H^+^ Concentration (mol L^−1^)
30	0.0140	3.98	3.70	2.02	1.81
50	0.0255	4.02	3.60	1.96	1.60
70	0.0353	3.95	3.35	1.89	1.40
90	0.0435	3.89	3.19	2.05	1.43

**Table 4:** Comparison between the real and theoretical proton concentration according to Faraday’s law.

Temperature (°C)	Current Density (A cm^−2^)	Theoretical Final H^+^ Concentration (mol L^−1^)	Experimental H^+^ Concentration (mol L^−1^)	10^3^ Non-Faradic Consumed Protons (mol)
30	0.0140	1.86	1.81	2.5
50	0.0255	1.66	1.60	3.0
70	0.0353	1.48	1.40	4.0
90	0.0435	1.54	1.43	5.5

## Data Availability

The data presented in this study are available on request from the corresponding author. The data are not publicly available due to privacy.

## Supplementary Materials

The following supporting information can be downloaded at: https://www.mdpi.com/article/10.3390/nano12081315/s1, Figure S1. EDX spectrum of the Pd/C electrocatalyst used in this study; Figure S2. XRD pattern of the Pd/C electrocatalyst; Figure S3. (a–c). TEM images with different magnification of the prepared 20% Pd/C, (d) Particle size distribution. References [29,61,62,63] are cited in Supplementary Materials.
