# Supplementary material for: An Alkaline-Acid Glycerol Electrochemical Reformer for Simultaneous Production of Hydrogen and Electricity
Source: Nanomaterials (Basel). 2022 Apr 12;12(8):1315. doi: 10.3390/nano12081315 (PMC9024791; doi:10.3390/nano12081315)
Supplement: Supplementary file 1 [file nanomaterials-12-01315-s001.zip › nanomaterials-1659377-supplementary.pdf]

*nanomaterials*

*Supplementary Materials*

# An Alkaline-Acid Glycerol Electrochemical Reformer for Simultaneous Production of Hydrogen and Electricity

**Fernando M. L. Amorim [1], Rudy Crisafulli [2] and José J. Linares [2,*]**

[1] Institute of Chemistry, Federal University of Goiás, Campus Samambaia, Avenida Esperança s/n, 74690-900 Goiania, Brazil; fernando.migueldelino@gmail.com
[2] Institute of Chemistry, University of Brasilia, Campus Universitário Darcy Ribeiro, 70910-900 Brasilia, Brazil; rudycrisafulli@gmail.com
* Correspondence: joselinares@unb.br; Tel.: +55-6131073901

Figure S1 shows the EDX spectrum of the prepared Pd/C electrocatalyst. Three spectra in different regions were applied, with an average Pd metallic weight percentage of 19.5 ± 0.8.

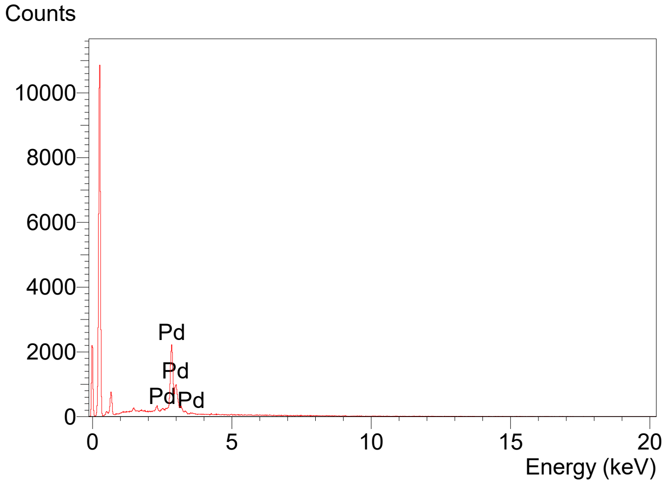


**Figure S1.** EDX spectrum of the Pd/C electrocatalyst used in this study.

Figure S2 shows the XRD pattern of the prepared electrocatalyst. Typical metallic Pd diffractions peaks are observed for Pd(111), Pd(200), Pd(220) and Pd(311) facets at 2θ angles of 40.1, 46.7, 68.1, and 82.1, respectively. The average crystalline ($D_{XRD}$) size corresponds to 5.0 nm after applying the Scherrer's equation (Equation (S1)) to the (220) peak [29], where 0.9 is the particle-shape dependent constant, $\lambda$ is the wavelength of the incidence radiation (Cu $K_\alpha$), $\beta_{2\theta}$ is the width in radians of the diffraction peak at half-height and θ is the half-angle of the selected peak.

$$D_{XRD} = \frac{0.9\lambda}{\beta_{2\theta}\cos\theta} \quad (1)$$

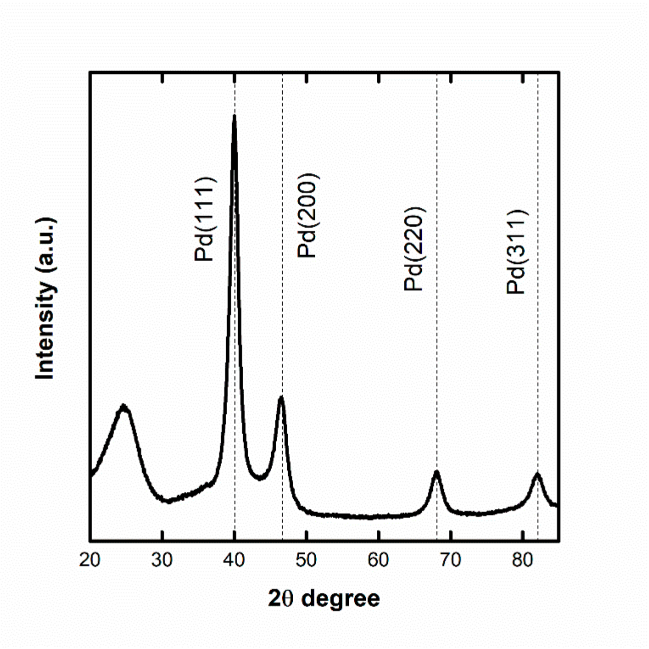


**Figure S2.** XRD pattern of the Pd/C electrocatalyst.

Figures S3a to c displays the TEM images of the prepared Pd/C. As can be seen, spherical Pd nanoparticles are distributed in a relatively homogenous way onto the carbon support, rendering an average particle size of 5.5 nm (Figure 2d). Preparation of Pd/C by the formic acid reduction method is not widely used, perhaps due to the tendency for form agglomerates [61–63] As a first remarkable feature, such large particulates are not observed in this proposed sodium formate reduction method. To try to understand this better, we are carrying out an ongoing discussion about a study that revealed a reduction in the particle size and agglomeration during reduction in strongly alkaline conditions (pH 13) and low sodium formate concentration (0.01 mol $L^{-1}$) compared to natural pH and higher formic acid concentrations.

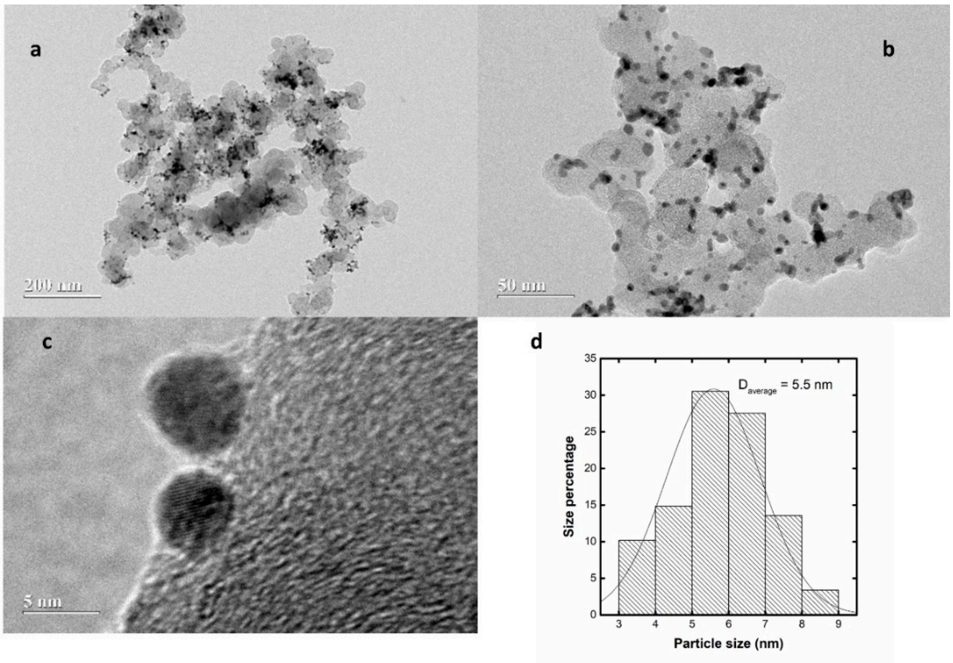


**Figure S3.** (**a**–**c**). TEM images with different magnification of the prepared 20% Pd/C, (**d**) Particle size distribution.
